# Proposed Early Cambrian cephalopods are chimaeras, the oldest known cephalopods are 30 m.y. younger

**DOI:** 10.1038/s42003-022-04383-9

**Published:** 2023-01-12

**Authors:** Ed Landing, Björn Kröger, Stephen R. Westrop, Gerd Geyer

**Affiliations:** 1 New York State Museum, 222 Madison Ave., Albany, NY USA; 2 Finnish Museum of Natural History, University of Helsinki, 00014 Helsinki, Finland; 3 Oklahoma Museum of Natural History and School of Geology and Geophysics, University of Oklahoma, Norman, OK 73072 USA; 4 Lehrstuhl für Geodynamik und Geomaterialforschung, Institut für Geographie und Geologie, Bayerische Julius-Maximilians-Universität Würzburg, Am Hubland, 97074 Würzburg, Germany

**Keywords:** Zoology, Physiology

**arising from** A. Hildenbrand et al. *Communications Biology* 10.1038/s42003-021-01885-w (2021)

Fossils record the lowest known occurrence of mollusc classes in the Cambrian Evolutionary Radiation (i.e., terminal Ediacaran–Cambrian, ca. 550–488 Ma). A very early occurrence of possible Early Cambrian cephalopods has been claimed^1^. However, these “cephalopod” fossils are a composite (a chimaera) with apertures of common septate orthothecid hyolith conchs with invaginated thin *Coleoloides* tubes misinterpreted as the diagnostic siphuncles of cephalopods. Cephalopods may have had an Early Cambrian origin, but their oldest undoubted fossils record an appearance and diversification as macropredators much later in the Late Cambrian and at the dawn of the Great Ordovician Evolutionary Interval.

**Fig. 1:**
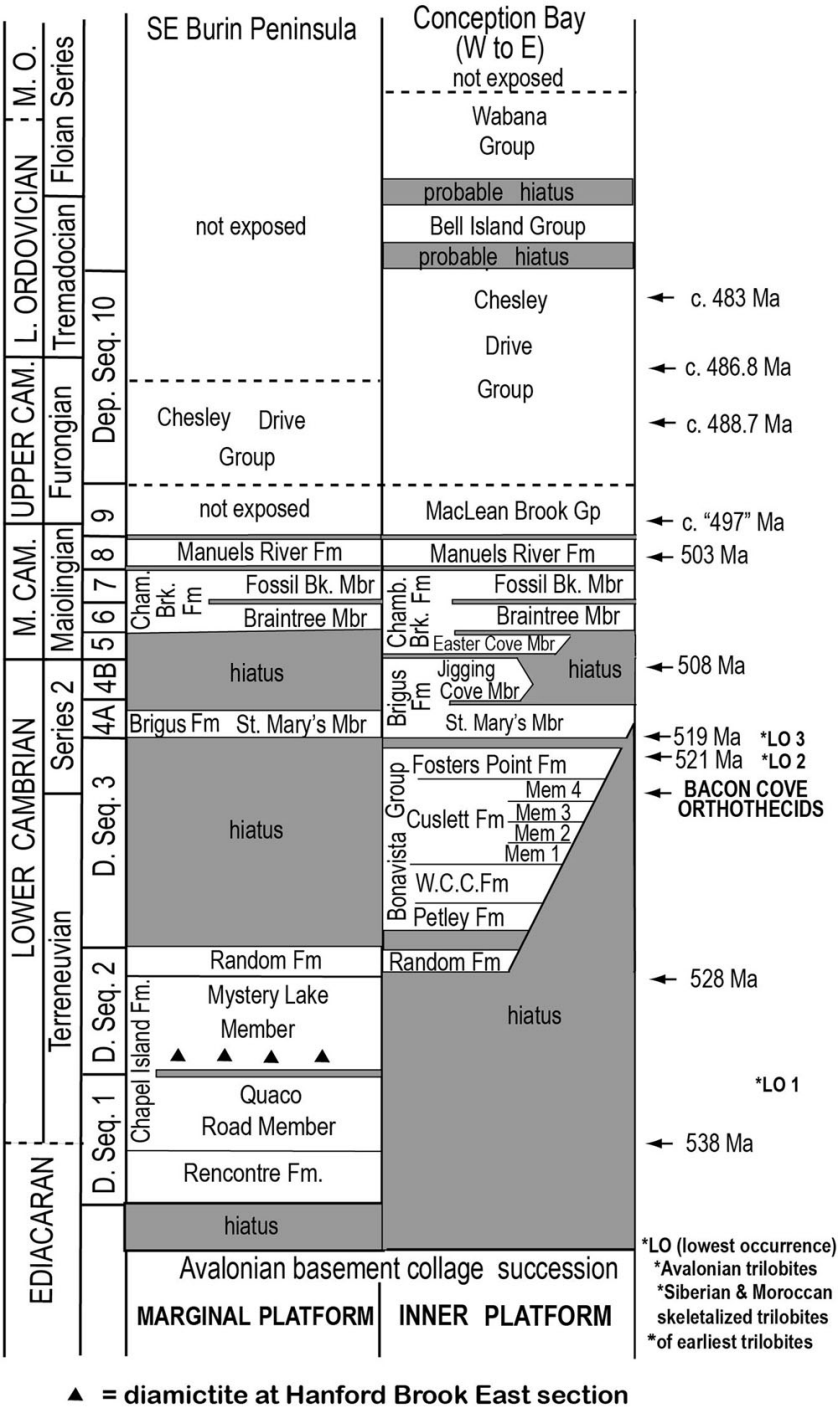
Stratigraphy of Avalonian SE Newfoundland. Terminal Edicaran–Lower Ordovician trans-Avalonian depositional sequences 1–10 in Burin Peninsula (marginal platform) and on east–west transect through Trinity–Conception bays (inner platform)^10–12^. Compare figure with Landing and Kröger^17^^, fig. 2^ (i.e., revised geochrology and sequence stratigraphy with post-Chesley Drive unconformity, shortened Dep. Seq. 10, diachronous onlap of Dep. Seq. 3 on inner platform, and reinterpreted basement geology). Dates from Landing et al.^6,7^. Bk. Brook, Cham. Chamberlain’s, D. depositional, Fm. formation, Gp. group, *LO lowest occurrences of trilobites discussed by Landing et al.^7^, Mbr member, M middle, O Ordovician, seq sequence, W.C.C. West Centre Cove.

Uncertainty surrounds the origin of cephalopods in early animal diversification. Did these marine predators appear in the Early Cambrian, as suggested by genomic study^2^, or do the oldest confidently identified cephalopod conchs record an origin and early diversification of major clades ca. 30 Ma later in the Late Cambrian^3–5^. Hildenbrand et al.’s^1^ interpretation of elongate calcareous conchs from SE Newfoundland seems to support an Early Cambrian origin. However, sufficient data are available in their report to allow comparison with existing reports on coeval elongate septate orthothecid hyoliths of their study locality and across SE Newfoundland and to re-evaluate of their specimens as a chimaera formed by post-mortem, wave-infill of orthothecid hyolith conch apertures with shell debris, including tiny tubular shells misinterpreted as the diagnostic siphuncles of cephalopods. (Lower/Early, Middle/Middle, Upper/ Late Cambrian are subsystems/subperiods^6,7^ (Fig. 1). They replace the undefined terms “lower”/”early”, “middle”/”middle,” and “upper”/“late” of many reports. Problems arise without unambiguous definition of these lower case adjectives; thus, an interval always assigned to the Middle Cambrian (Drumian) is “upper Cambrian” in a high distribution journal^8^.)

Hildenbrand et al. ^1^ describe Early Cambrian “cephalopods” from Bacon Cove, SE Newfoundland (Fig. 1). Bacon Cove has an earlier described^9^^, fig. 35^ Cambrian sequence that is part of the inner platform of the ancient Avalonian microcontinent^10^. In this area, the Cambrian was deposited in strike-slip basins and unconformably overlies a basement collage of Meso- and Neoproterozoic blocks^10–12^. Redefinition of Lower Cambrian stratigraphy at Bacon Cove and across SE Newfoundland^8^, not followed by Hildenbrand et al.^1^^, fig. 5b^, includes a lower mudstone-dominated unit (Member 4 of the Cuslett Formation, Bonavista Group). The eroded, Ediacaran at Bacon Cove is locally covered by ca. 2.0 m of quartzose sandstone of  basal Member 4. A thin stromatolitic bed on the highest Ediacaran is overlain by a thin red limestone with purported “cephalopods.”

Depositional and diagenetic features of the red limestone are key to understanding its “cephalopods.” The limestone has abundant, elongate conchs (3 cm long) and other calcareous fossils that form a packstone with a fine grained microsparite matrix^1^^, fig. 1a, b^. Avalonian Cambrian microsparites show early aggradational recrystallization of lime mud^13^. Skeletal fragments in these successions were originally mainly aragonite, and are now replaced by calcite spar. Original calcite sclerites occur in a few taxa (i.e., trilobites) and retain original microfabric. Few taxa had phosphatic sclerites (tommotiids, lingulates), and there is negligible phosphatization by comparison with coeval Siberian and South China facies^13,14^.

At Bacon Cove, we recorded^9^ a roughly bimodal (east–west) orientation of the elongate conchs consistent with wave action e.g., ref. ^15^. Wave-dominated deposition of Avalonian peritidal packstones led to complex depositional histories with burial, exhumation, and reburial of fossil debris that may leave internal cavities (i.e., conch apertures) empty to completely filled with lime mud. Post-mortem invagination of fossil fragments occurs in sclerite apertures e.g., refs. ^1^^, fig. 1a;^^16^^, fig. 7.2^. Early diagenetic calcite spar can partly fill open cavities and lime mud can then fill the lumen by infiltration or following exhumation and reburial of a conoidal fragment e.g., refs. ^1^^, fig. 2^. These depositional and early diagenetic events are shown by Hildenbrand et al.^1^^, figs.1, 2a, 4^.

The Bacon Cove conchs have been compared^1^ with those of the orthothedid hyolith “*Allatheca*” *degeeri*, which has fewer, more widely spaced septae e.g., ref. ^16^. A closer comparison is with the conchs with variable cross sections of the orthothecid “*Ladatheca*” *cylindrica*, which are abundant in Avalonian peritidal facies^14^; have closely spaced septae; and smaller apical angle^17^. These features are comparable to the facies of and features of the conchs in Hildenbrand et al.^1^.

The evidence is that the Bacon Cove “cephalopods” include elongate conchs of the common “*Ladatheca*” *cylindrica*. This species has such hyolith (and non-cephalopod) features as an operculum^16^^, fig.9.2^ and a fusiform protoconch (cephalopods have cap-like protoconchs^17,18^) in its syntypes^16^^, fig. 9.8^.

Bacon Cove “*Ladatheca*” *cylindrica* conchs have apical septa. Septa are taxonomically non-diagnostic and occur in different animal groups in sclerites (e.g., lapworthellids, other tommotiids) and conchs (i.e., orthothecid and hyolithid hyoliths, gastropods, cephalopods)^19^. Septae reduce the volume of energy-demanding soft tissue^16,19^ and do not necessarily show homology with septae used in cephalopod buoyancy control e.g., ref. ^17^.

The defining features in ancestral cephalopods are a conch with an open aperture (living chamber) and posterior phragmocone^19^. The latter is defined by a siphuncle—a skeletal structure that perforates and connects the septae^3^. Hildebrand et al.^1^^, figs. 2a, 4b^ illustrate tubular structures interpreted as “submarginal siphuncles” with “connecting rings”. However, there is no evidence these tubes connect and perforate septa—a precondition for interpretation as a siphuncle, with perforate septa unknown in the Bacon Cove material.

The “siphuncles” are best interpreted as invaginated (cone-in-cone), sediment-filled, small conchs comparable to those in the “*Ladatheca*” *cylindrica* conch matrix^1^^, fig. 1a, b^. Invaginated conchs with sparry calcite fill indicating reworking of diagenetically altered (“pre-fossilized”) conchs occur in an “*L*.” *cylindrica* conch aperture^1^^, fig. 1a, b^—with the septate specimen on the right comparable to “*L*.” *cylindrica*. The tiny conch on the left is likely referable to that of *Coleoloides typicalis* e.g., ref. ^16^—which are about same size and have a circular cross section as the sediment-filled “siphuncles”^1^^, figs. 2, 4b^.

Hildenbrand et al.^1^ note submarginal “siphuncles”—which are not located very close to the ventral conch wall which would be more consistent with early cephalopods^3–5^. Far more concerning is that one of the “siphuncles” is not longitudinal but inclined^1^^, fig. 4b^, as shown by a more elongate cross section than the large conch it is in. This is expected in an inclined, invaginated tube.

Another feature known in cephalopod conchs are cameral deposits—calcareous shell material deposited on the interior chamber walls and septa of some conchs. Hildenbrand et al.^1^^, figs. 1d, 2c^ claim to illustrate cameral deposits on the inner wall of the “phragmocone” of the Bacon Cove material. However, the posited “cameral deposits” are not distinguishable in their very dark figures, while it must be noted that cameral deposits are unknown before the Ordovician e.g., ref. ^3^.

This evidence is that the Bacon Cove “cephalopods” are chimaeras—an association of several taxa, none of which is a cephalopod. Although “holotype” and “paratypes” of an unnamed taxon are named and figured^1^^, Supp. Fig. 1^, these are type specimens of a chimaera, not a biological organism. In their tentative cephalopod assignment, Hildenbrand et al.^1^ describe these chimaeras in terms specific to cephalopods (i.e., phragmocone, siphuncle, connecting rings)—which means an a priori taxonomic assignment colors their specimen descriptions.

Absence of evidence is used as evidence for a cephalopod assignment of the Bacon Cove specimens by Hildenbrand et al.^1^. Thus, lack of crossed lamellar microhistology of septa is said to argue against a hyolith assignment^1^, but replacement of hyolith aragonite by calcite spar and loss of original microhistology is characteristic in Avalonia and in our Bacon Cove specimens^6^. Indeed, hyolith microhistology is only known from phosphatized, not calcite replaced, specimens^20^. Similarly, lack of an operculum supposedly precludes a hyolith assignment^1^; which really means Hildenbrand et al.^1^ did not find the readily detached “*L*.” *cylindrica* operculum^21^.

Finally, phosphatic “siphuncles”/”connecting rings” (i.e., the *Coleoloides*? conchs) is claimed to support a cephalopod assignment of the Bacon Cove material^1^. However, this is incorrect; the common phosphatic connecting rings of Phanerozoic cephalopods are not a taxonomic feature but reflect secondary diagenesis of organic-rich connecting rings^22–24^. The phosphate is difficult to interpret in the Bacon Cove material: An EDX image of a 1.2 mm wide, purportedly double-walled, phosphatic “connecting ring” is figured^1^^, fig. 3c, d^, but a double-wall is absent in an optical image of the same(?) “ring,” which is only 0.5 mm wide^1^^, fig. 2^.

In conclusion, Early Cambrian conchs from SE Newfoundland are not cephalopods. They are a chimaera composed of conchs of the common orthothecid hyolith *“Ladatheca” cylindrica* with some apertures containing invaginated *Coleoloides* tubes. Correlation of the uppermost Fosters Point Formation (Fig. 1) shows the chimaeras are older than the lowest Siberian trilobites^25^. However, the lowest occurrence of trilobites is very diachronous, and they have a probable early Terreneuvian origin^26^, a proposal supported by Bayesian analysis of trilobite evolution^27^. In short, the Bacon Cove material is far younger than the earliest euarthropods *contra* Hildenbrand et al.^1^. The oldest definite cephalopods are even younger (ca. 30 m.y.), with Bayesian phylogenetic analysis showing a middle Late Cambrian–earliest Ordovician (Tremadocian) diversification of the class Cephalopoda into three major clades (subclasses) and several unassigned orders^5^. An earlier fossil record of the class remains unknown, with the Bacon Cove conchs confidently referable to the Hyolitha and not to the cephalopods. The middle Late Cambrian diversification of predatory cephalopods and appearance of diverse and abundant euconodonts marked the modernization of marine ecosystems and dawn of the Great Ordovician Diversification Interval^13,26^.
